# Dental Hints Department

**Published:** 1906-06

**Authors:** B. H. Teague

**Affiliations:** Aiken, S. C.


### DENTAL HINTS DEPARTMENT

**Conducted by B. H. Teague, D. D. S., Aiken, S. C., to whom all contributions to this department should be sent.**

A superior quality of tooth picks is now manufactured from swamp reeds.—*Pop. Mech's.*

Black lead and suet, equal parts, mixed, make a good jewelers' rouge.—*Popular Mechanics.*

A very satisfactory soft solder consists of a mixture of 2 parts tin and 1 part lead.—*Pop. Mech's.*

Pressure Anesthesia.—In pressure anesthesia amadon is superior to rubber for a covering as it takes up, instead of allowing to escape, the adrenalin and cocain.—*Mr. Hey, British Dental Journal.*

General Anesthetics.—I maintain that until the degree of D. D. S. gives us the privilege of signing a death certificate, we have no right to deal with general anesthetics.—*Dr. DeC. Lindsley, Dental Era.*

To keep plaster of Paris from hardening so quickly mix it with vinegar instead of water.—*Gordon M. Backus, Popular Mechanics.*

If a mandrel becomes so rusted or worn that the screw no longer holds, a light blow with a hammer will flatten the hole slightly and cause the threads to hold as good as ever.—*Dr. A. C. Wittman, Review.*

Dental Anaesthetic and Lunacy.—Harry S. Strickler, a prosperous milkman of York, Pa., disappeared on January 8, and his family and relatives claim he has been driven insane by an anæsthetic taken in a dentist's office.—*Philadelphia Record.*

To Remove Grease, Paint, etc., from Metal.—To remove grease, paint, etc., from machinery, add half a pound of caustic soda to two gallons of water and boil the parts to be cleaned in the fluid. It is possible to use it several times before its strength is exhausted.—*Machinery.*

Pyorrhoea Alveolaris.—A line of "continuous, mild medication" which has proven efficacious consists in placing silver bands about the roots of the teeth, to furnish silver salts for their typical effect.—*W. V. B. Ames, Dental Review.*

Saddle Bridges.—Contrary to the generally accepted belief that a saddle is decidedly unhygienic, such a device is frequently indicated in order to obtain a *closer approach to an hygienis result.—Hart J. Gosslee, Dentists' Mag.*

Recipe for Violet Aniline Ink.—Dissolve 1 oz. of the best violet aniline in 4 oz. hot alcohol. When thoroughly dissolved add 1 gal. boiling water. The cost wil be about 60 cents.—*Pop. Mech's.*

Alaska tin, it is prophesied, has a great future before it. It is now being smelted on a small scale at Seattle, Wash., and this smelter is soon to be enlarged to handle tin ore concentrated at Teller, Alaska.—*Exchange.*

Coffee is a good antidote for acetanilide. This should be mentioned to patients when prescribing it, as it may prevent much harm, and save life.—*Dr. J. A. Burnett, Med. Brief.*

A small amount of acetanilide put in the hollow of an aching tooth will quickly relieve it. Guaiacol is superior to acetanilide for toothache, and should always be used in place of acetanilide, when on hand.—*Dr. J. A. Burnett; Med. Brief.*

An unfortunate Parisian the other day happened to call for professional assistance on a dentist who was insane. The dentist pulled out eight of his patient's teeth, quieting his objections with a revolver. Afterward he shot two policemen while being arrested.—*Newspaper.*

To Preserve Rubber.—One part of ammonia in ten to twelve parts of water will preserve soft rubber. Dip rubber pipes, etc., in a glass jar filled with this solution. For the ammonia bottle use a rubber stopper; it is better than a glass one.—*Cosmos.*

The Three Principal Advantageous Features of Porcelain over Gold.—First, its quality as a preservative of the teeth; second, its application is effected with much less exhaustion to both patient and operator; third, its almost ideal harmonious effect in matching the natural teeth.—*Henry G. Raymond, Dental Register.*

Time to Apply Borax.—Apply the borax and solder before heating, and heat as far as possible over a good flame before applying the blow-pipe.—*L. P. Haskell, Dentists' Mag.*

The Bandless Crown.—If in fitting the bandless porcelain crown to the root, the same care is taken as in fitting a band, the operator can reproduce the natural crown of the tooth in every way, a crown as near perfect as any crown can be.—*U. M. Richardson, Dental Review.*

Nervousness.—Often due to autoxemia, by which the blood is not sufficiently oxygenated. Calomel and soda, one grain of each at bedtime for a few nights, often works wonders. If a nerve sedative is indicated, a preparation of celery in proper doses will be found the least harmful.—*Dr. W. T. Marrs, Med. Brief.*

Retention of Dentures.—If possible two roots should be left in each jaw and utilized to support the plate. A satisfactory method is to fit each such root with a gold cap and tube, into which fit a pin attached to the plate. The stability which even one root so treated will give to an entire denture is surprising.—*Wm. M. Gabriel, Dental Record.*

Death From Alveolar Abscess.—A young man, who had always been delicate, complained of toothache and it was found that an abscess had formed in the jaw. This was lanced and morphia was injected, but the young man suddenly collapsed later on and died. "Œdema of the glottis, due to cellulitis of the neck, consequent upon a decayed tooth," was the verdict of the Coroner's jury.—*Dental Record.*

Supporting a Sore Tooth While Drilling.—Instead of supporting a sore tooth by ligature to prevent pain while it is being drilled, take modeling compound, soften it and make a splint for both lingual and buccal sides of the teeth to support the sore tooth while drilling. This will prevent jarring and also prevent pressure on the inflamed peridental membrane.—*T. L. Gilmer, Dental Review.*

Sodium Dioxid.—Sodium dioxid is chiefly employed in dentistry as a bleaching agent, but will, through its alkaline and caustic properties, obtund sensitive dentin. The only safe way to use sodium dioxid for either purpose is to make a saturated solution in water, as much heat is generated, sometimes accompanied by ignition, when the dry powder is brought in contact with moisture in a tooth.—*Brief.*

Good Old Fashioned Things.

Old fashioned work, old fashioned rectitude,
Old fashioned honor and old fashioned prayer,
Old fashioned patience that can abide its time,
Old fashioned firesides sacred from the world,
Old fashioned satisfaction with enough,
Old fashioned candor and simplicity,
Old fashioned folk that practice what they preach.
—*The Young Catholic Messenger.*

The Porcelain Inlay.—The fact that porcelain has a greater range of application, is more permanent, more compatible, harmonizes in color better, is more sanitary, and requires less physical exertion upon the part of both patient and operator than any other material, will force its universal adoption in time.—*F. E. Roach, Dental Digest.*

John Roach Spooner deserves a place in dental history as one who discovered that valuable agent, arsenic, for pulp devitalization, and also for his early research and experiment in perfecting the usefulness of porcelain teeth.—*Brief.*

Gold Crown Location.—It of course is the opinion of nearly all of us that the uses of gold crown farther forward than the first molar or second bicuspid are rare indeed, and often in these cases we can with good results bring into use the porcelain facing.—*D. A. House, Dentists' Mag.*

To Clarify Shellac Varnish.—Even with the best of care the pattern maker will find his shellac leaving dirty streaks on the pattern from various impurities held in suspension in the varnish. These may be entirely precipitated by the gradual addition of some crystals of oxalic acid, stirring the varnish to aid this solution, and then setting it aside over night to permit the impurities to settle. No more acid should be used than is really necessary.—*Machinery.*

Locating Foreign Bodies in the Throat.—In cases of foreign body in the throat never neglect to ask the patient, if sufficiently intelligent, to point with the finger to the site of the greatest pain and discomfort, as this affords a valuable clue to the position of the foreign body or of the abrasion inflicted. If situated above the pharyngeal constrictors the patient is apt to put his finger into the mouth, while if more deeply seated he will indicate some place on the outside of the throat.—*International Journal of Surgery.*

To repair a gold crown that has a hole worn through it, drill out the hole, parallel the side walls with good cement, make a matrix of platinum and make a gold inlay. Cement to place and finish.—*Dr. H. H. Gantz, Albia, Iowa.*

Now if we step into the laboratory, we can save time and gas by attaching to our Bunsen burner an ordinary by-pass. It affords a ready and convenient means of turning the flame up or down at a moment's notice.—*Dr. A. C. Wittman, Review.*

Cementing Inlays.—To maintain pressure on inlays in proximal cavities until the cement crystallizes, spring a piece of nursing bottle tubing between the teeth. Allow it to remain until the cement has set.—*Oliver Martin, Review.*

"My business belongs to me twenty-four hours a day. I belong to my business from nine till six o'clock. From six till nine I must be free. I can do more work by thus restricting my hours of business, and I believe I can do better work in those hours."—*Exchange.*

Opening Large Cavities.—Employ a chisel and hand mallet to break down enamel walls. A slight tap will accomplish what would otherwise require much hand pressure, and without so much danger of the chisel slipping and injuring the soft tissues.—*Oliver Martin, Review.*

When a root about to receive a shell crown is a little too short to afford a good hold, a very small brass wood screw screwed into the canal will give you a feeling of security. The projecting head gives the cement a grip it otherwise would not have.—*Dr. A. C. Wittman, Review.*

Treatment for Canker Sore Mouth.—I have found the full strength aromatic sulphuric acid almost a specific for this condition. I prescribe internally tincture of ferric chloride gtt. v.; potassium chlorate gr. iii.; water ½ oz. every three hours in lemonade.—*J. E. Power, The Tri-State Dental Journal.*

"We use Dr. Jenkins' new porcelain enamel, or prosthetic porcelain, which fuses just under the fusing point of pure gold, and is exceedingly strong and fine grained. It unites perfectly with the English teeth and can be ground and repolished as circumstances and convenience require. We consider it the *ne plus ultra* of strength, reliability, and convenience of manipulation."—*Dr. J. A. Spaulding, Dentists' Mag.*

The Gold Matrix: Protection From Fusing.—It is not necessary that any matrix be invested; when gold is used it is protected from fusing by coating it, preferably with rouge because of its great fineness and affinity for a smooth surface. The rouge is bought in powder and spatulated with alcohol and water. It will resist heat to an astonishing degree.—*W. A. Capon, Items of Interest.*

The investment is of more importance than the flux. The investment and porcelain conduct heat slowly, while all of the metals are good conductors. The investment and porcelain must be heated in advance of the metal, then there will be no danger of fracture of the porcelain.—*Dr. S. H. Voyles, Brief.*

Protective for Porcelain During Soldering.—When circumstances make it necessary to near-by soldering to invest crowns or bridges porcelain-face up, cover the porcelain with thin asbestos paper saturated with the investment mixture, catching the free ends in the body of the investment proper. The paper protects throughout the operation from the direct action of the flame.—*Office & Lab.*

In waxing up plates it is very convenient to have some melted wax where you can dip into it at any time. A small tin cover may be riveted to a wire frame in such a manner as to keep it at the proper height above your Bunsen flame. The one I have is kept three and one-half inches above the flame, and at that height the wax is kept melted and yet does not boil over.—*Dr. A. C. Wittman, Review.*

Mixing Amalgams.—When an alloy is amalgamated and the excess of mercury removed by squeezing, an unknown quantity of the constituents of the alloy is removed by the mercury, depending upon the solubility of the metals in mercury. To obtain the best results they should be mixed with a definite quantity of mercury, and under no conditions must excess of mercury be used, and the excess to be removed by squeezing.—*F. J. Brislee, Dental Record.*

To Repair a Broken Tooth in a Gum Section.—Grind out the broken tooth from the section, even with the gingival margin, then select a plain vulcanite tooth of proper size and shade and grind to fit the space. Pack in fresh vulcanite around the pins of the tooth and vulcanize as an ordinary repair case. If a little care has been used in making the joint at the gingival line, the job cannot be detected from a full new block.—*F. H. Wilkinson, Dental Summary.*

Interdental Splints.—The interdental splint—either vulcanite or metal—is the most universally applicable splint, for with slight modifications it may be successfully applied to fractures of either the maxilla or the mandible, and to those involving any portion of the mandible from the symphysis to the neck of the condyle.—*H. DeW. Cross, Dentists' Mag.*

Advice to Patients.—There is only one way in which I can bring people to appreciate the value of cleanliness in the mouth, and that is to tell them that they would not sit down at a table to eat with knives and forks that were one-tenth as dirty as their own teeth are, and still they have their teeth in their mouth all the time. That comes nearest of anything I have found to bringing them to their senses.—*E. A. Royce, Review.*

Rest and Recreation.—In the hum and hustle for business we must not forget the world. Ye need not only rest, but recreation, and the resources are many, viz.: Spending an evening with a neighbor talking over current events, attending a social gathering, attending church and many other things too numerous to talk of, but which will benefit you and yield satisfactory returns if you do not use them merely to advertise your business.—*S. A. Helch, Dental Review.*

Narcotile Anaesthesia.—The beauty about narcotile anaesthesia is its pleasantness. Patients are insensible to pain long before they are past talking. I can go ahead and operate, the patient being almost entirely conscious but feeling slight or no pain. I have given narcotile and removed temporary abscessed teeth for almost babies who would find no objection save that "that stuff made their ears roar." The patient always recovers completely in about five minutes and there are no after effects.—*W. H. Reaben, Practical Dental Journal.*

Treating Root Canals.—For a number of years in the general practice of treating root canals in which abscesses had been formed the chemical agents used were various preparations containing principally the volatile oil series, which, as is well known, have no special power of disinfecting, and really have but little value as antiseptics. They are extremely irritating to the tissue cells, and many times sufficiently stimulating to the bacterial forms of life as to produce cell proliferation in those organisms, thus producing an increased number of bacteria instead of diminishing them.—*Dr. G. W. Cook, Era.*

Sensitive Cavities.—For sensitive cavities previous to excavating, I have found an application of the following very effective: Zinc iodide crystals, 1½ grs.; iodide crystals, 2 grs. Make a solution of this in glycerine. Wind a small pellet of cotton on the end of a broach, dip it in the solution and apply it to the decay. For removing the stain use peroxide of hydrogen.—*E. M. S. Fernandez, Review.*

A safe formula for abscesses without a fistula is:

B

Tricresol, f3j (gm. iv),

Formalini, f3ss (gm. ij). M

Sig.—Mechanically evacuate the pus and, on cotton, hermetically seal in the canals from twenty-four to forty-eight hours, two or three times. Oftentimes one treatment is sufficient.—*Dr. J. P. Buckley, Dentist's Mag.*

Woman's Dental Association.—The annual meeting of the Woman's Dental Association was held at 1716 Chestnut street, Philadelphia, March 3, 1906: Dr. H. Belle Whitcomb presided. The following officers were elected for the year 1906–1907: Emily W. Wyeth, president; H. Belle Whitcomb, vice-president; Rebecca R. Cornish, corresponding secretary; Eliza Yerkes, recording secretary; Elizabeth A. Davis McDonald, treasurer. Executive Committee: Frances Crouch, Annie L. Locht, Martha Corkhill, Anna K. Leaming, Matilda Grath.—*Eliza Yerkes, Secretary, Philadelphia, Pa., Brief.*

A National Dental Law Wanted.—A movement to obtain a national dental law, such as will permit dentists who have met standard requirements to practice in any state in the Union, was launched at the thirty-eighth annual meeting of the Seventh District Dental Society of Now York. The action followed the reading of a paper on "Dental Legislation and Interstate Comity," by Dr. W. A. White, of Phelps. In his paper Dr. White made the suggestion that a national examining board be formed, from which all dentists prepared to do so might receive a diploma entitling them to practice in any state in the country. There was much discussion of the question, and the resolution that follows was adopted: "Resolved, That a committee be named to confer with the state society, with a view to the appointment of a member from each state society, all of whom shall meet in an interstate convention and formulate a uniform interstate dental law."—*Brief.*

Good Cleansing Polish.—A good encaustic, which will clean and polish at the same time, is composed of 1 gallon soft water, 4 oz. yellow laundry soap and 1 lb. of white wax, shaved up. Boil together, stirring well, and then add 2 oz. sal soda; put the mixture in something which can be closely covered, and stir constantly until cool. If necessary dilute with water before using; lay on with a paint brush and polish off with a hard brush or cloth. Can be used on furniture, marbles, tiles and bricks. Will remove ink stains.—*Popular Mechanics.*

Nitrate of Silver.—For ten years I have made it a rule to see that all the teeth were given a good treatment with a saturated solution of nitrate of silver, as soon as possible after eruption. I simply dry off the surfaces and put on the solution with a small swab, letting it stay a minute, during which time I push it with an explorer down into the sulci. I give this as an invariable rule, carried out with great success for the above length of time. Decay is generally prevented, or when it does occur is greatly delayed.—*Harry F. Hamilton, International Dental Journal.*

Comprehensive.—The following is the copy of a sign exhibited over the door of a general store in a little out-of-the-way Cornish village, and reads: "R. G. ———, Surgin. Parish Clark, Groser and Undertaker, respectably informs ladys and gentlemen that he drors teeth without wateing a minut, apply leches every hour, blisters on the lowest terms, and visicks for a penny apiece. As times is crul bad I begs to say that I have beginned to sell all sorts of stastionary ware, cox, hens, fowls, pigs, and all other kind of powltry. I have also laid in a large assortment of trype, lollipops, ginger beer and matches, and other pikkles, such hepsom salts and winzer sope."—*Dom. Jour.*

Business Committees' Supplies.—Who has not heard the cry at a dental meeting: "Where's the blackboard?" "Anybody got a piece of chalk in his pocket?" "If I had a tack or pin, Mr. President, I could hang this chart where it might be seen to advantage." But the blackboard has been left in somebody's attic, no good man goes about loaded with chalk, tacks are not thought of, and the pin that comes to the rescue is pointless and badly bent. Some things ought not to be; these are of those. Wherefore, let the chairman of the business committee, or the man who attends to his business for him, provide for all possible needs of those who may take part in the doings of the society.—*Office & Lab.*

On the Manipulation of Cements.—In mixing cements, be quick and active. There is a tendency in the profession to mix cements too thick. Get away from that tendency; get away from it as far as possible. The kneading or working with the fingers of a mixture of dental cements prior to the introduction into a cavity is all wrong. Fill your cavity flush, and let it alone. Don't constantly disturb it till you cannot disturb it any more on account of the advanced stage of crystallization. If you agitate plaster of Paris while it sets it gets crumbly. Please apply this fact to dental cements. If you introduce dental cement in the right consistence it will exhibit a glossy surface when set.—*D. Maurice Albrecht, Dental Summary.*

Obtunding Sensitive Dentine.—1. I use the high pressure syringe.

2. I am, and with the most gratifying results to my patients and myself.

3. I do not explain it. I simply know it does the work and as yet, I have seen no harmful results.

4. I think it does, if pressure is continued long enough. I do not anticipate any evil results when intelligently used.

5. Its action is much quicker and is of course safer for you know where your cocain is going.

I would like to say farther, that I consider this method of obtunding sensitive dentine, one of the very greatest helps which has come to us in recent times.—*Dr. A. J. Sawyer, Dentists Mag.*

To assist in maintaining an easy and comfortable position I have been in the habit, for some months, of using an ordinary desk stool when operating upon the superior arch, and find that I can thus avoid putting myself into the shape of a corkscrew, as was often necessary when trying to perform the same operations in a standing posture. I now use the sitting posture in fully eighty per cent of all operations upon the superior arch, and find myself at the close of a day's steady work very much less fatigued than formerly. I usually bring the tooth to be operated upon either parallel to or a little above the eye as I sit.

A trial of this plan will be all that is necessary to make an operating stool a permanent fixture in a dentist's office.—*Dr. H. C. Register, Brief.*

Sanitary Bridgework.—That a fixed bridge is never indicated I am not prepared to say, but that the removable bridge has a wider field, with greater possibilities and many advantages, I am thoroughly convinced. We have incomparably a more sanitary denture, greater possibilities of restoration of the lost tissues, whereby more natural and artistic results are obtained. Repairs are made easily and any subsequent repairs upon adjoining teeth are more simplified and less destruction of natural teeth is required for abutments. Another advantage of no small importance is the fact that good, serviceable and artistic bridges may be made with vulcanite at a moderate cost, but the ideal material for this class of work is porcelain.—*F. E. Roach, Review.*

Professional Man or Mechanic.—A learned judge of Tennessee has recently made a great effort to determine to which class the dentist belongs, and leaves the matter as unclarified as he found it. How long will it take people of intelligence to discover the true basis of professionalism? All dentists are mechanics, so are all surgeons. All dentists, however, are not professional men, neither are all surgeons or lawyers or clergymen. A professional man is one who gives his life to the pursuit of his art or calling with unselfish devotion to those whom he is to serve, using every means at his command to perfect himself, both technically and scientifically. Under this definition few qualify, but all can who will.—*Editorial Register.*

An Operating Stool.—The position to be assumed by the dentist in many operations upon the teeth is not a small matter when the length of time he is required to maintain it is taken into account.

If the position is an unfavorable one, the result is backache, chest pains, nervous prostration, dyspepsia, sleeplessness, and, if too often repeated, a premature grave.

Besides, in order to give the patient the benefit of all the attention and concentration of thought, and the best manipulative skill of which one is capable, it is necessary that the operator should himself be in comfortable circumstances. A constrained or painful position must, therefore, necessarily detract very materially from the desired result.

